# NeuroTest: a benchtop testbed for evaluating sensing-capable electrophysiology and neurostimulation systems

**DOI:** 10.1088/1741-2552/aeb82d

**Published:** 2026-11-04

**Authors:** Morrow JK, H Cho, M Benjaber, Denison TJ, Widge AS, Herron JA

**Affiliations:** 1 Department of Psychiatry and Behavioral Sciences, University of Minnesota, Minneapolis, MN, Unites States of America; 2 Department of Neurological Surgery, University of Washington, Seattle, WA, Unites States of America; 3 Institute of Biomedical Engineering, University of Oxford, Oxford, United Kingdom

**Keywords:** deep brain stimulation, electrophysiology, neuromodulation

## Abstract

*Objective*. The development of increasingly complex implantable neuromodulation devices requires extensive benchtop validation and testing prior to *in vivo* implementation. Our goal is to enable this *in vitro* testing by creating a benchtop system that is accessible, easy-to-use, and functions reliably across academic and industry environments, reducing discordant results that arise from discordant approaches. Furthermore, such a system paves the way for standardizing benchtop characterization of future implantable systems by establishing a repeatable and comparable methodology for validating these devices and their capabilities. *Approach.* We describe the NeuroTest Board (NTB), a low-cost, open-source device that combines a low-noise current source waveform generator with a high-precision data acquisition system. We provide information on its architecture, operation, and performance as an initial benchmark of the potential capabilities of the system. *Main results.* We demonstrate the use of the NTB to assess electrophysiology systems in two separate applications. In one case, we assess a commonly used laboratory system (the RHD2132 digitizing headstage and Open Ephys data acquisition system), while the second case characterizes an investigational neuromodulation device in development for human use (the CorTec Brain InterChange). Following this initial sensing characterization, we explore stimulation-related artifacts that would hamper *in vivo* data collection. With the NTB, we develop and evaluate artifact mitigation strategies for each system, thus demonstrating an example application of this benchtop testbed. *Significance.* The ability to test neuromodulation devices against a common benchmark will enable faster development of novel therapeutics by increasing inter-institutional reliability and decreasing troubleshooting time.

## 1. Introduction

As the field of neuromodulation research grows, there is a complementary rise in increasingly complex devices that expand the features and research capabilities available to scientists. Electrode arrays used for sensing complicated neural dynamics continue to increase in density [1–3] and diversify in terms of materials and form factors [2, 4, 5]. Advances in the designs of electrodes for electrical stimulation have enabled more sophisticated control of current delivery [6–10]. These technological developments have led to novel therapeutic interventions, including adaptive stimulation paradigms that track patient-specific neural activity in real-time to guide stimulation delivery (for review see [11]). These novel closed-loop and adaptive paradigms have been applied in Parkinson’s Disease [12, 13], epilepsy [14], depression [15–17], and pain [18, 19].

The confluence of advances in hardware, software, and conceptual frameworks underlying neuromodulation-based therapies has created exciting opportunities to generate further breakthroughs in treating neurological and psychiatric disorders [20–24]. However, leveraging these developments to create safe, reliable, and easy-to-operate systems presents many serious challenges (see [23, 25] for overviews). Laboratory electrophysiology systems are notoriously susceptible to noise from sources such as wall power supplies, digitization noise and aliasing, and even from other equipment within the same experimental setup [26–28]. Even implanted, fully battery-backed systems can be susceptible to interference from strong signals of no physiologic interest [29]. Another nearly ubiquitous issue within the field of closed-loop neuromodulation is mitigation of stimulation artifacts (e.g. [30–32] or see [33] for review). After these issues are sufficiently resolved, researchers still need to thoroughly assess how their system handles realistic electrophysiological signals of interest. These troubleshooting steps are crucial for ensuring proper system function but can be time consuming and costly.

While there are currently a variety of tools available for denoising electrophysiology systems, mitigating stimulation artifacts, or simulating complex neural activity, these methods feature limitations and/or gaps in function that impede the comprehensive assessments needed for system development (table 1). To address this problem, we have created the NeuroTest Board (NTB), an open-source benchtop tool that enables more thorough testing of electrophysiology equipment. Though the NTB was designed with broad testing purposes in mind, it is especially useful for assessing closed-loop neuromodulation systems designed to work with field potential data (e.g. local field potentials or electrocorticograms). Here, we describe the NTB in terms of design and function and describe a pair of example use cases for mitigating electrical stimulation artifacts. We start by first detailing some of the currently available methods for assessing electrophysiology systems, highlighting limitations that are resolved by the NTB.

**Table 1.** Comparison between general features of currently available commercial signal generators, *in vivo* models, and an ideal testbench. *High fidelity sensing* refers to how well a system can faithfully capture weak electrical signals. Most commercial and proprietary signal generators lack sensing circuitry and are therefore marked as not applicable (N/A) while basic oscilloscopes (not show) do not come with signal generation capabilities. *Output resolution* refers to the size of the voltage or current steps of the signal generator, determined by the bit count of the system driving the output. *Amplitude range* is the range in peak-to-peak units that a system can output. While these ranges can generally be altered via external voltage dividers, we list typical values natively supported by each category of signal generating system. *Neural emulation* refers to how well a system can model neural data in terms of complexity (e.g. from simple functions like sine waves to fully customizable waveform generation or replay of neural data), amplitude, and emulation of the tissue electrode interface. We note that while some waveform generators in the ‘Standard Signal Generator’ category can replay pre-recorded data (e.g. the Keysight 33 500 series listed in datasheet [34]), the typical inability to drive <1 mVpp outputs without external voltage dividers makes them suboptimal for emulating neuroelectric activity. Animal model testing features real neural data and is therefore listed as ‘True.’ *Responsiveness* refers to how well a system can react to events in real time. In some cases, Standard and Proprietary signal generators can make use of external triggers, such as TTL pulses, to update their output. This is a useful feature but requires integration of external systems that may further complicate the testing setup or introduce new sources of noise. The first generation of the NTB described in this manuscript enables simple reactivity via EP emulation. An ideal testbench would further expand on this capability to enable more complex responses (see Discussion). *Accessibility* encompasses all barriers to usage, including overall cost, necessary infrastructure, and access to code, designs, and data. Lastly, we note that a comparison of systems built solely for biopotential playback (i.e. signal generators designed to emulate neuroelectric activity but that lack responsive circuitry) can be found in Powell *et al* 2021 [35].

	Standard Signal Generators e.g. [34, 36, 37]	Proprietary Signal Generators e.g. [38–40]	Bespoke Signal Generators e.g. [35, 41]	*In Vivo* Animal Models	NeuroTest Board	Ideal testbench
High fidelity sensing	N/A	N/A	N/A	N/A	High	High
Output resolution	High 16+ bit	High 16+ bit	Very High 24 + bit	N/A	Very High 24 bit	High 16+ bit
Output amplitude range	1 mVpp to 10 Vpp	10 *µ*Vpp to 10 mVpp	∼1 *µ*Vpp to >10 mVpp	<1 *µ*Vpp to >10 mVpp	∼1 *µ*Vpp to >10 mVpp	∼1 *µ*Vpp to >10 mVpp
Neural emulation	Poor	Moderate to High	High	True	High	High
Responsiveness	None/Low	None/Low	None	True	Moderate	High
Accessibility	Low to Moderate	Moderate	High	Low	High	High

### 1.1. Limitations of current benchtop methods

#### 1.1.1. General purpose signal generators

Benchtop tools such as oscilloscopes and signal generators can provide high fidelity data acquisition and high resolution, fine-grained control over signal outputs. These features are critically important for testing electrophysiology equipment; however, the ability of commercially available tools to evaluate innovative neuromodulation systems and adaptive algorithms is limited. Traditional broad-purpose laboratory equipment, such as generic waveform generators, are generally unable to replicate neural signals in terms of complexity and amplitude. For example, many basic signal generators are limited to function driven waveforms (e.g. sine or square waves) and/or are unable to output signals below 1mV (see datasheets in [36, 37] for examples). These issues make them suboptimal for emulating the microvolt-level, highly complex oscillations seen in local field potential (LFP) or electrocorticogram (ECoG) recordings [42]. More advanced, broad-purpose signal generators designed to mimic biopotentials are available (e.g. [43]); however, they can cost over R10 000. High price points limit accessibility, especially for early development when projects may have limited funding.

#### 1.1.2. Proprietary neuroscience-specific signal generators

Some proprietary signal generators [38–40] created by neuroscience-focused companies have addressed these issues by adding the functionality to play back simulated neuroelectric activity at low-voltage ranges. However, they typically present a limited set of signals available for playback that may not model the electrophysiological characteristics of the intended *in vivo* application (e.g. Digital Neural Signal Simulator from Blackrock Neurotech superimposes a few simple sinewaves to emulate LFP data). These proprietary systems are also often purpose-built around interfacing with a limited range of other devices, typically ones made by the same manufacturer or a limited number of commercial partners. While some vendors may be able to supply end-to-end support for entire experimental paradigms, cutting-edge science often involves incorporating new technologies from multiple sources (see Perspective in [23] or examples in [44, 45]). Reliance on a single vendor or part can also lead to issues if critical parts are discontinued or supply chains are disrupted (as noted in [4]).

#### 1.1.3. Bespoke testbenches for neuromodulation research

To expand on the features provided by these commercial solutions, more customizable biopotential simulators [35, 41] and hardware-in-the-loop testing frameworks [46] have been introduced that provide multichannel, low-noise signal playback. These benchtop tools enable customized signal generation, including replay of previously recorded neural activity, which greatly expands the types of test signals available to better capture the specific target application of interest. However, the testbench solutions in [35, 41] were primarily designed to evaluate the sensing capabilities of neuromodulation devices either through direct voltage injection or a resistive load setup. This type of circuit fails to account for the effects of the tissue-electrode interface and thus, the impact of stimulation on this interface and the sensing circuit. This is a critical oversight. The nervous system functions as an electrolyte medium in which ions serve as the charge carriers [47], as opposed to the electrons that flow in resistive loads [48]. The electrode-electrolyte interface adds an inherent capacitance and impedance [49, 50] as well as the potential for reduction-oxidation chemical reactions if voltage windows are exceeded [51–53]. Both phenomena can filter and distort both applied stimulation waveforms and recorded tissue responses. These issues indicate that testing in an electrolyte, such as a saline bath, better mimics *in vivo* conditions relative to a resistive load setup. Indeed, Powell *et al* 2021 performed a set of tests of their Neuro digital-to-analog converter (DAC) system via saline bath and noted the differences between the ‘Wired’ and saline setups [35]. Finally, testing within saline setups is considered standard procedure for medical devices under the International Organization for Standardization [54].

#### 1.1.4. Closed-loop neuromodulation

Each of the various signal generation systems listed thus far also lacks one key element for assessing potential closed-loop neuromodulation systems: responsiveness. Real neural systems respond to external events, including electrical stimulation, with rapid physiologic responses. These responses may depend on the state of the nervous system at the time of stimulation [55–57]. Some responses, such as evoked potentials (EPs), serve as biomarkers for assessing connectivity [58–61], neural excitability [55, 62], and could be used to assess or improve the effectiveness of neuromodulatory interventions [63, 64]. Sensing an EP is dependent on a multitude of factors, including: (1) Uncertainty in whether the stimulated tissue can respond to the provided stimulation, e.g. due to refractory periods or the amount of current delivered. (2) Aspects of the electrophysiology system, such as sensing configurations and stimulation artifact affecting the detection of an EP, e.g. long amplifier settling times obscuring neural responses to stimulation [31]. (3) Signal processing techniques, which have been demonstrated to affect the final EP measurement and yield notable differences in the final EP signal [65]. The lack of means to separate the effects of signal processing from system-related characteristics can result in ambiguity as to whether a system under test can reliably extract biomarkers of interest. Resolving those ambiguities requires that the testing tool itself be a closed-loop system that can sense a device’s delivery of stimulation and respond with physiology-like activity. None of the tools listed in sections 1.1.1–1.1.3 can achieve this.

### 1.2. Challenges with *in vivo* models

The issue of emulating biological complexity, including the responsive nature of nervous tissue, can be addressed via the use of animal models. Indeed, *in vivo* testing via animal models is an important step in the development of breakthrough treatments both in terms of biocompatibility assessments and scientific investigation. But research animals also require significant investment and infrastructure (i.e. purchasing costs, per diems, housing, investigator care/husbandry, etc). Additionally, animal model testing often requires species-specific customization, for example, downscaling the size of a device, power supply, or connectors relative to the ultimate clinical design [25, 66, 67]. Such changes can limit the translation of results from animal models to clinical application. Furthermore, *in vivo* conditions can introduce factors that may complicate system troubleshooting. It is difficult, or even impossible in some cases, to distinguish which aspects of incoming data are driven from a specific source of interest (e.g. neural activity in a target brain area) versus those that are a function of environmental noise, non-neural activity, or activity from a neural source the researcher is not interested in [26, 32, 33, 68–70].

### 1.3. Summary of design needs

A novel testing tool would combine the strengths of the above methods while minimizing their shortcomings. Based on these strengths and weaknesses, we identified the following Needs (summarized in table 1) as criteria for the NTB:

(1) Bidirectional neural interface: to properly test the bidirectional neural interfaces used in closed-loop neuromodulation equipment [30, 71–73], the test system should be bidirectional. It must s*ense low-amplitude signals with high fidelity*, in order to denoise a system under test, and g*enerate precisely controlled low-amplitude outputs* that serve as ground truth for testing.
(2) Neural emulation: the testbench must be able to accurately generate signals that precisely mimic neural activity. The system should specifically be able to replay recorded neural data or advanced simulations of neural data. Signal output should be designed for use in an electrolyte medium to properly emulate the ion flux of *in vivo* systems.
(3) Responsiveness: the testbench should detect and react to real time events with millisecond latencies to mirror the time course of neural responses to electrical stimulation. This requires that it must also sense stimulation coming from a device under test.
(4) Accessibility: the system should be accessible in terms of price as well as access to designs, code, and data. An open-source system will more readily facilitate widespread dissemination and adoption of benchmarks and standards compared to proprietary systems.

## 2. Neuro test board overview

We created the NTB to address these needs. The NTB is an open-source, low-cost *in vitro* benchtop testing system (figure 1(A)) for assessment of electrophysiologic tools, especially those for neuromodulation research. The platform pairs sensing circuitry with biopotential signal playback to create a system capable of reacting in real time to simulate the brain’s response to changing events, states, or stimulation. We demonstrate how the platform can be used to emulate EPs driven by electrical stimulation and show how this functionality can be used to test the capabilities of both well-characterized and investigational devices used in neuromodulation research. These features will help clinicians and engineers be better informed about the capabilities of proposed study devices prior to approaching *in vivo* experiments. With the NeuroTest platform, the aim is to accelerate the process of designing and validating bidirectional implantable interfaces and enable the creation inter-laboratory standards for assessing system functionality.

**Figure 1.**
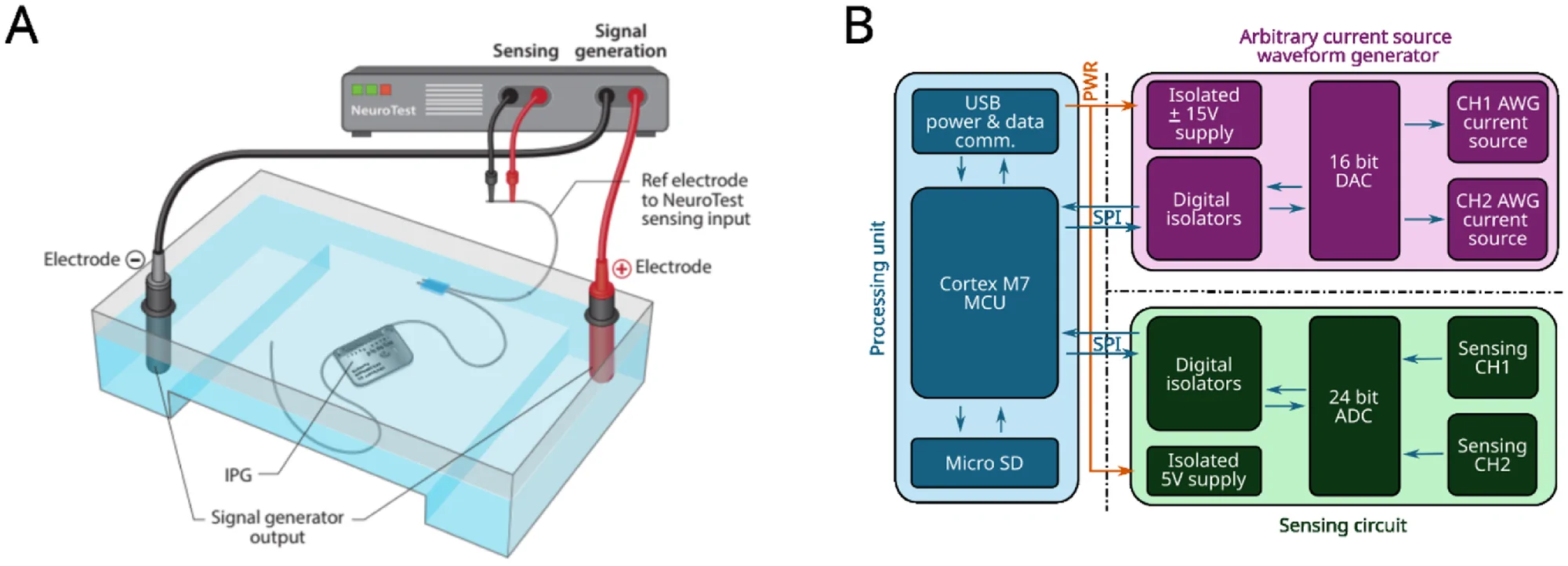
Overview of the NeuroTest Board platform. (A) A cartoon depiction of the general use case of the NeuroTest Board (top) connected to a gel electrophoresis tank and a submerged implantable pulse generator (IPG) (bottom). (B) Block diagram depicting the components of the NTB. The system is divided into three components: the processing unit (blue), the waveform generator (purple), and the sensing circuit (green). Power lines (PWR) are shown in orange while communication between the components is shown by the blue arrows. Serial peripheral interface (SPI) cables are used for bi-directional communication between the processing unit and the sensing circuit and waveform generator.

### 2.1. NTB design

The NTB uses a stripped-down gel electrophoresis chamber (BioRad Inc) filled with a 0.9% saline solution in which the system under test is submerged. While there is no international standard phantom for testing closed-loop neuromodulation devices according to the implanted device standard ISO 14 708 [54], use of an electrophoresis tank allows for application of a stable and even electrical field across the test medium. This consistency is crucial for repeatability and these tanks provide an accessible commercial product that many labs will be familiar with, making them a reasonable option for the testing medium. The tanks feature banana-plug connectors that are attached to wires submerged in the saline, which function as a cathode and an anode. Connecting these to a signal generator allows for voltage or current to be injected across the entire tank.

The electronics of the NTB were designed to generate simulated biopotential signals, such as those measured via LFP or ECoG recordings, while simultaneously sensing the output of a neural device under test. The NTB includes three main components: (1) a processing unit, (2) a precision arbitrary current source waveform generator (ACSWG), and (3) a precision sensing circuit. These three components of the NTB are galvanically isolated from each other to reduce noise, interferences, and ground loops between the different circuit functions. A system diagram of the NTB is illustrated in figure 1(B) and additional technical schematics of its components are shown in figures 1(C) and (D). A full list of parts and circuit designs are included in the Supplemental Materials.

#### 2.1.1. Processing unit

The first section of the NTB contains the microprocessor, a 32-bit Arm Cortex M7 clocked at 600 MHz, which is programmed to be controlled and powered with a USB 2.0 connection that enables communication speeds up to 480 Mbps. We opted to use this processor as it can easily handle reading two channel 24-bit analog-to-digital converter (ADC) data (used for signal acquisition, see section 2.1.3), write to two channel current source signal generators (see section 2.1.2), read data from an external source (e.g. SD card), and stream data to the control computer at full USB bandwidth (i.e. 480 Mbps). Importantly, this microprocessor provides additional headroom for implementing/expanding future functionality (see the Discussion section). The Cortex M7 processor is based on the development board Teensy 4.1, which allows for user-friendly, accessible programming via the open-source Arduino integrated development environment (IDE). This section of the board also includes a microSD card that allows users to load neural data for replay. This microSD card can also be used to save data observed during tests with the NTB, though data can also be saved directly to a control computer via the USB interface. The NTB is powered by a 5 V supply via the USB connection. This connection also allows the user to update firmware on the NTB via the open-source Arduino IDE. Likewise, data can be streamed to the connected computer and visualized in the Serial Plot tool from Arduino or other data visualization software. The precision ACSWG and the precision sensing circuits are both powered from the same source as the processing unit, using two galvanically isolated DC-DC converters. One ±15 V supply powers the ACSWG circuit, while the sensing circuit is supplied by an isolated 5 V supply. All communication between the processor unit and the other two components is established using digital isolators.

#### 2.1.2. Precision ACSWG

The NTB has two independent low-noise ACSWGs based on a Howland current source architecture [74] (figure 1(C)). The Howland current source was used to provide a precision bipolar current source over a wide dynamic range. It offers high output impedance, which helps maintain a controlled output current even when the impedance of the test medium or electrode interface changes. These features, along with the relatively simple circuit topology, make the Howland current source a good fit for our purposes. While the selected resistors are anticipated to be with 1% of matching, we note that resistor mismatch would result in reduced output impedance. However, a feedback capacitor is included that helps with stability and resistors are taken from the same reel and lot to minimize variation in impedances. The current source circuit is designed to generate currents in the range of 0–10 mA with a 305 nA resolution driven by an ultra-low noise 16-bit DAC combined with a low-noise, ultra-low offset amplifier. Because the sensing applications of a device under test will be dependent on electrode impedance and position of the recording dipole, the range and precision of the NTB’s ACSWG were chosen to allow a user to mimic a myriad of neuro-electric phenomenon in the scales observed *in vivo* [42]. For instance, LFP signals observed with current-generation sensing-capable implants tend to be in the 1–200 *µ*V range [75, 76], and *in vivo* stimulation EPs in the 100–1000 *µ*V range [77, 78]. The ACSWG has a voltage range of ±14 V supplied from low-noise linear regulators (LDOs) to enable the system to maintain the required current delivery over a large range of impedances depending on the *in vitro* setup used. The 305nA output resolution of the ACSWG ensures that the NTB has sufficient flexibility to emulate low-amplitude signals across different experimental environments, including those with higher impedances.

The bandwidth of the current source is reduced from 1 MHz to 100 kHz using a feedback capacitor to reduce the noise introduced by the NTB when connected in saline. Specifically, inadequate anti-aliasing by a device being tested can cause high frequency noise to fold back from the current source, distorting the sensing capabilities of the test device. We programmatically set the generator to produce activity up to 2000 Hz by default to enable simulation of a wide range of neural oscillatory events [42], but this range can be changed by an end user as needed. In this work, we use the NTB to inject signals that lack power in frequencies above 200 Hz, making an output rate of 2000 Hz sufficient for high fidelity rendering of our signals of interest. However, all users should consider tailoring the output frequency of the ACSWG to produce high fidelity signals according to their specific needs in alignment with the Nyquist theorem.

The output of the ACSWG can be calibrated to produce voltages that are within expected ranges for the specific recording setup of an end user. That is, the amount of current being output is scaled such that the voltage values detected by a downstream data acquisition system (the ‘realized voltage’) are in the appropriate ranges when considering electrode size and material, dipole location, and other elements of the recording arrangement. This calibration is achieved via a function made available in the user interface. The function works by injecting sine waves into the saline tank at progressively increasing amplitudes via the ACSWG, which are then measured by the sensing component of the NTB. As part of the calibration process, the NTB automatically adjusts the gain of the output such that the sensed values more closely match the original sine wave input generated.

#### 2.1.3. Precision signal acquisition

The NTB features a two-channel, high-precision data acquisition circuit with a 24-bit resolution delta-sigma ADC supplied by an isolated 5 V DC-DC converter. The data acquisition circuit also offers a configurable sampling frequency up to 32 kHz, and configurable gain from 1 to 128 V/V. A 24-bit ADC was selected to provide the required dynamic range and sensitivity for biopotential signal acquisition (i.e. microvolt level RMS), while also accommodating larger input signals and stimulation artifacts without saturation when required. This ability to tune the data acquisition circuit enables flexibility in biopotential sensing, as a gain of 1 V/V provides a wide input range (±1.2 V) with an input-referred noise floor of 4.25 *µ*V RMS at a 2 kHz sampling rate while a gain of 32 V/V reduces the usable input range (±37.5 mV), but improves the theoretical noise floor to 1 *µ*V for the same 2 kHz sampling rate [79]. Further, the ability to programmatically configure the gain stage provides a secondary method to avoid saturation from stimulation artifact. This is achieved by lowering the gain when expecting or assessing large stimulation levels, which is coupled with the high common-mode rejection ratio of the sensing front-end. Lastly, we note that the sensing circuit includes built-in clamping diodes to protect the front-end during delivery of electrical stimulation. These limit the input voltage to a safe range and allow the sensing circuit to recover quickly once the stimulation artifact has passed.

The precision sensing circuit has a programmable high pass filter that allows the user to filter out DC offsets and low frequency noise. The range of the high pass filter is dependent on the sampling frequency set of the ADC for the NTB. For example, with a 2 kHz sampling rate, the high pass filter can be configured between 4 mHz–90 Hz. The precision sensing circuit is controlled via the processing unit through digital isolators such that the sensing circuit is fully isolated from the other two parts of the NTB, reducing noise and crosstalk. While the ADC sampling frequency of the NTB can be programmatically set up to 32 kHz, we used a sampling rate of 1 kHz to 1) match the lowest sampling rate among the data acquisition systems being assessed in these specific tests (i.e. the CorTec Brain Interchange (BIC) system, described below), 2) because the data used in this work has little high frequency power (i.e. >200 Hz), and 3) because the majority of bioelectrical field phenomena feature spectral profiles in the 0.1–500 Hz band [42, 80, 81]. It should be noted that while this sampling rate is appropriate for our uses according to the Nyquist theorem, other users should set this value to match their specific needs.

## 3. Benchtop validation of NTB functionality

Benchtop assessments were performed to evaluate the functionalities of the NTB. A diagram of potential use setups of the NTB is visualized in figure 2. Considering that neuromodulation systems have many use cases, we designed the NTB to adapt to a wide variety of applications. To demonstrate this versatility and reliability, two distinct benchtop environments, each at different laboratory sites, were configured for assessments for site-specific applications. Testing at the first site focused on evaluating the performance of an Intan RHD2132 amplifier that was paired with the OpenEphys data acquisition system (figure 2(A)). These components were central pieces of equipment for experiments that use animal models and we therefore refer to this setup as evaluating an animal-use research (AUR) system. Work at the second site was intended to characterize the investigational CorTec BIC system (figure 2(B)) [82–84]. This device was designed for use in human patients, and we refer to this site setup as evaluating a human-use research (HUR) system. In both the AUR and HUR setups, NTB testing aimed to assess the sensing capabilities of the systems during stimulation, with the goal of culminating in robust signal processing pipelines for characterizing cortico-cortical connectivity and developing closed-loop paradigms. The decision to utilize the BIC system in particular was driven by a recent investigational device exemption granted by the FDA for use in an ongoing clinical study [85].

**Figure 2.**
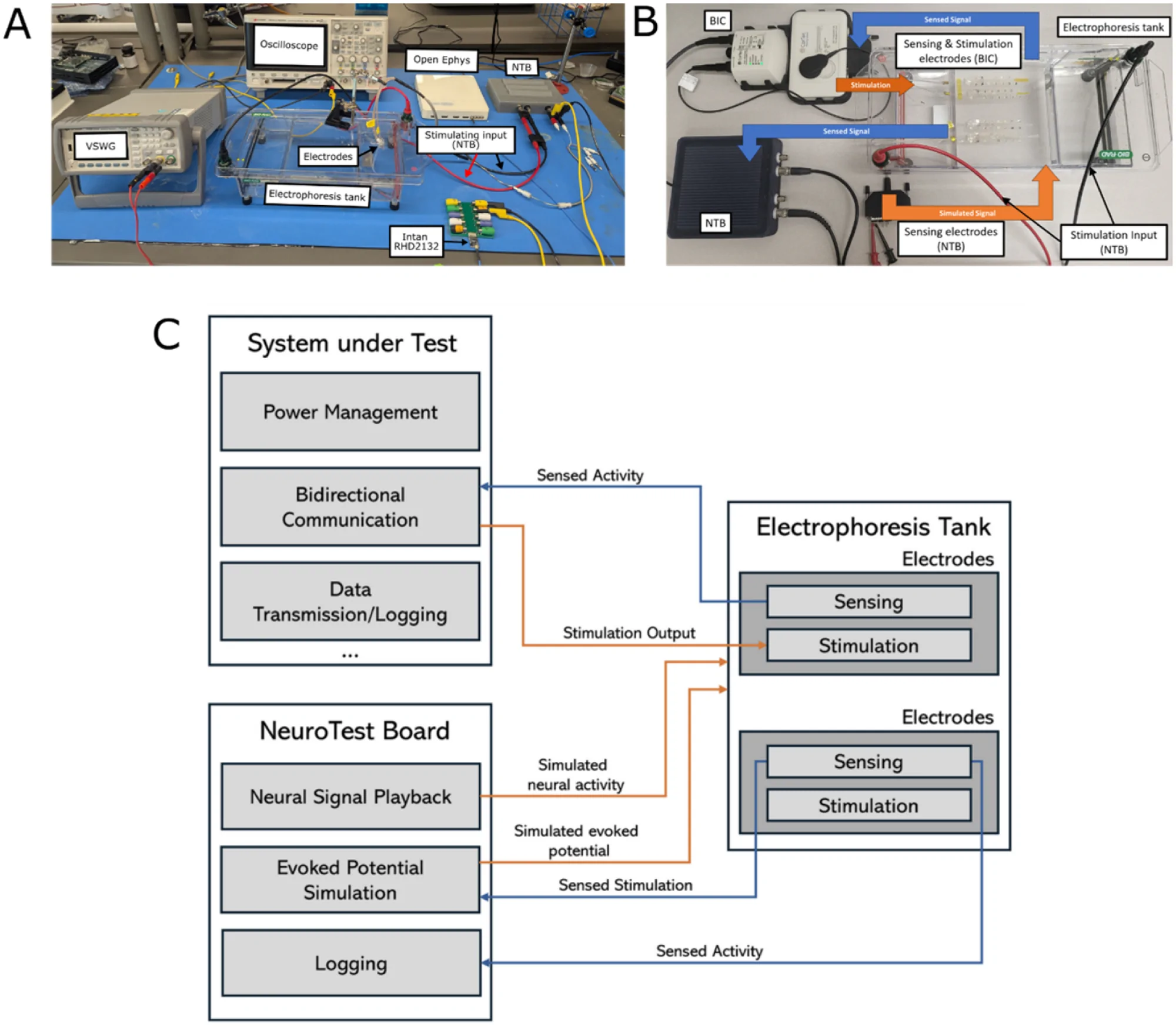
Benchtop environments incorporating the NeuroTest Board. (A) Picture of the benchtop setup intended to support the animal-use research (AUR) setting. A Keysight 33500B voltage source waveform generator (VSWG) and a Keysight oscilloscope (DSOX2004A) were used to verify proper NTB function. Up to six electrodes (Pt–Ir, 125 *µ*m diameter, 1–20 kΩ) submerged in 0.9% saline are used for sensing or stimulation. Signal from the same electrodes can be split and routed to multiple systems. Here, we show data being routed to both the NTB (directly) or an Intan RHD2132 headstage (via a breadboard, see yellow and black cables) using banana plugs. Data from the RHD2132 is then routed to an Open Ephys data acquisition system. An external electrical stimulator (e.g. StimJim, not shown) was connected to the electrodes when testing electrical stimulation pulse delivery. The waveform generator of the NTB is connected across the cathode and anode of the tank. (B) Picture of the benchtop setup intended to support the human-use research (HUR) setting. Sensing and stimulating electrodes from the CorTec Brain InterChange (BIC) device are submerged in 0.9% saline in the electrophoresis tank. The sensing side of the NTB is connected to two electrodes from an identical sEEG array using a modified D-sub connector. The waveform generator of the NTB is connected to the cathode and anode of the tank. Colored arrows indicate the flow of signals between the BIC and NTB. Blue arrows reflect the sensing feature of both systems, and the orange arrows reflect the stimulation output generated by both systems, highlighting their bidirectional nature. (C) Block diagram showing a generic setup using the NTB and a closed-loop system under test such as the CorTec BIC. Colored arrows visualize the flow of information between the different components either into or out of the testing medium (i.e. the saline tank). Note that the signal generator of the NTB is shown as connected to the saline tank (i.e. via the cathode and anode connectors on the exterior of the tank) in this diagram but it can also be connected to sensing or stimulation electrodes in submerged in the tank.

### 3.1. Devices under test

#### 3.1.1. AUR benchtop environment

In the AUR setup (figure 2(A)), between six and twelve platinum-iridium electrodes (built in-house using 125 *μ*m diameter wire from AM Systems, 1–20 kΩ impedances) were submerged in the 0.9% saline bath. These electrodes were wired to banana plugs, which were used to connect to various downstream devices. Simple banana-to-BNC adapters were used to connect electrodes to the sensing side of the NTB, a StimJim electrical stimulator [86], or a Keysight digital oscilloscope (DSOX2004A, Keysight Technologies). A splitter was used to route parallel signals from the NTB sensing channel to a custom banana plug breakout board. This board was used to connect banana plugs to a 32-channel Omnetics connector, which in turn allowed the signal to be routed through the Intan RHD2132 headstage. The headstage was then connected to an Open Ephys data acquisition system (DAQ) [87] via a serial peripheral interface cable. Either a Keysight voltage source waveform generator (33500B, Keysight Technologies) or the precision ACSWG of the NTB was connected to the cathode and anode of the electrophoresis tank using banana-to-banana plug connectors. Both the oscilloscope and voltage source waveform generator provided parallel means to cross check that the various components of the AUR setup were functioning as expected (e.g. we checked that the NTB, Open Ephys, and oscilloscope all registered sine waves injected via the NTB or waveform generator at the expected amplitudes and frequencies).

The Intan RHD2132 used in these tests features a 16-bit ADC and 32 unipolar amplifiers with integrated analog filters. We shorted the reference and ground pins together and verified that all headstages used were fully functional (i.e. no dead channels or unusual noise characteristics).

#### 3.1.2. HUR benchtop environment

The HUR benchtop environment, depicted in figure 2(B), was comprised of the BIC system connected to two sets of 32 channel ECoG grids submerged in a saline solution. An additional ground electrode, an optional lead hard-wired to the implant casing connected to the ECoG grids, was also submerged in saline. For the sensing reference, a distant electrode relative to the sensing channel on the ECoG grids was selected. A single set of ECoG electrodes consisted of two grids of 2 × 8 platinum-iridium (Pt–Ir) circular contacts and ended in a 37-pin D-Sub connector. A set of ECoG grids were connected to the BIC and NTB each. While the D-Sub connector directly connected to the implant module of the BIC development kit, additional connectors were used to connect the NTB to specific pins of the D-Sub connector. This connection scheme made it such that only one channel was used for sensing with the NTB, but otherwise replicated the sensing configuration used with the BIC. The output from the NTB was connected to the cathode and anode of the saline tank. However, the ground electrode was not shorted to any contacts on the ECoG grid. These channels were not shorted together during subsequent testing to more realistically reflect noise characteristics likely to be found *in vivo* conditions.

### 3.2. Process for characterizing noise and artifact

To characterize system noise of the NTB, electrodes connected to the NTB and either the Intan RHD2132 or BIC were submerged in a saline solution, using the setups shown in figure 2. For these noise tests, a single sensing channel on the NTB was connected across a single pair of recording and reference electrodes that were submerged in the saline. Prior to recording, the NTB underwent calibration, as described in section 2.1.2, to ensure measurement reliability. Once calibration was completed, the NTB and both systems under test were configured to record while no input was injected into the saline bath. After obtaining 2-minute-long recordings with the above setup, RMS noise and the noise floor were determined. To compute noise floor values, power spectral densities (PSDs) were calculated via Welch’s method, using a 0.5 s-long Hamming window and 50% overlap for the recording channel, and a P_ref_ = 1 *µ*V^2^ Hz^−1^.

A segment of these raw recordings is shown in figures 3(A)–(D) to illustrate typical recordings from the NTBs. Despite distinct laboratory locations and setups, the NTBs showed minimal noise fluctuation across sites (figures 3(A)–(F)). Maintaining a gain of 1 for both NTB systems, RMS values for the NTB were calculated across the entire 2-minute recordings, yielding values of 4.06 *µ*V (AUR) and 4.01 *µ*V (HUR). After establishing consistency in the noise characteristics of the NTB between sites, the NTB was used to assess the noise floors of the amplifiers unique to each testing site. PSDs were calculated for the setups at each site using activity captured from the NTB and systems under test (figures 3(E) and (F)). The NTB showed less line noise (60 Hz and harmonics) than the Intan/Open Ephys data (figure 3(E)) as well as lower noise floor at all frequencies relative to the BIC (figure 3(F)). Due to differences between bandwidth recording frequencies for the NTB (1–1000 Hz), Intan (1–7500 Hz) and BIC (1–500 Hz), the PSDs for each of these recordings reached a noise floor plateau at different frequencies. The PSDs determined at the HUR site showed the BIC plateauing around 500 Hz at −4 dB with the NTB settling near 1000 Hz at around −24 dB. At the AUR site, the PSD for the NTB also settled near 1000 Hz at −24 dB while the Intan setup settled near 600 Hz at −25 dB. Lastly, we note that the use (or not) of a 60 Hz notch filter was dependent on the system being tested. In the AUR setup, a 60 Hz notch filter was not used because the Intan RHD2132 does not include hardware filters for line noise and the programmable notch filter from the Open Ephys DAQ is not enabled in our default configuration. A notch filter is natively present in the BIC data acquisition system. Therefore, we opted to include a notch filter in the NTB data used for assessing the HUR setup but not for the AUR setup to better mirror the devices being tested in each case.

**Figure 3.**
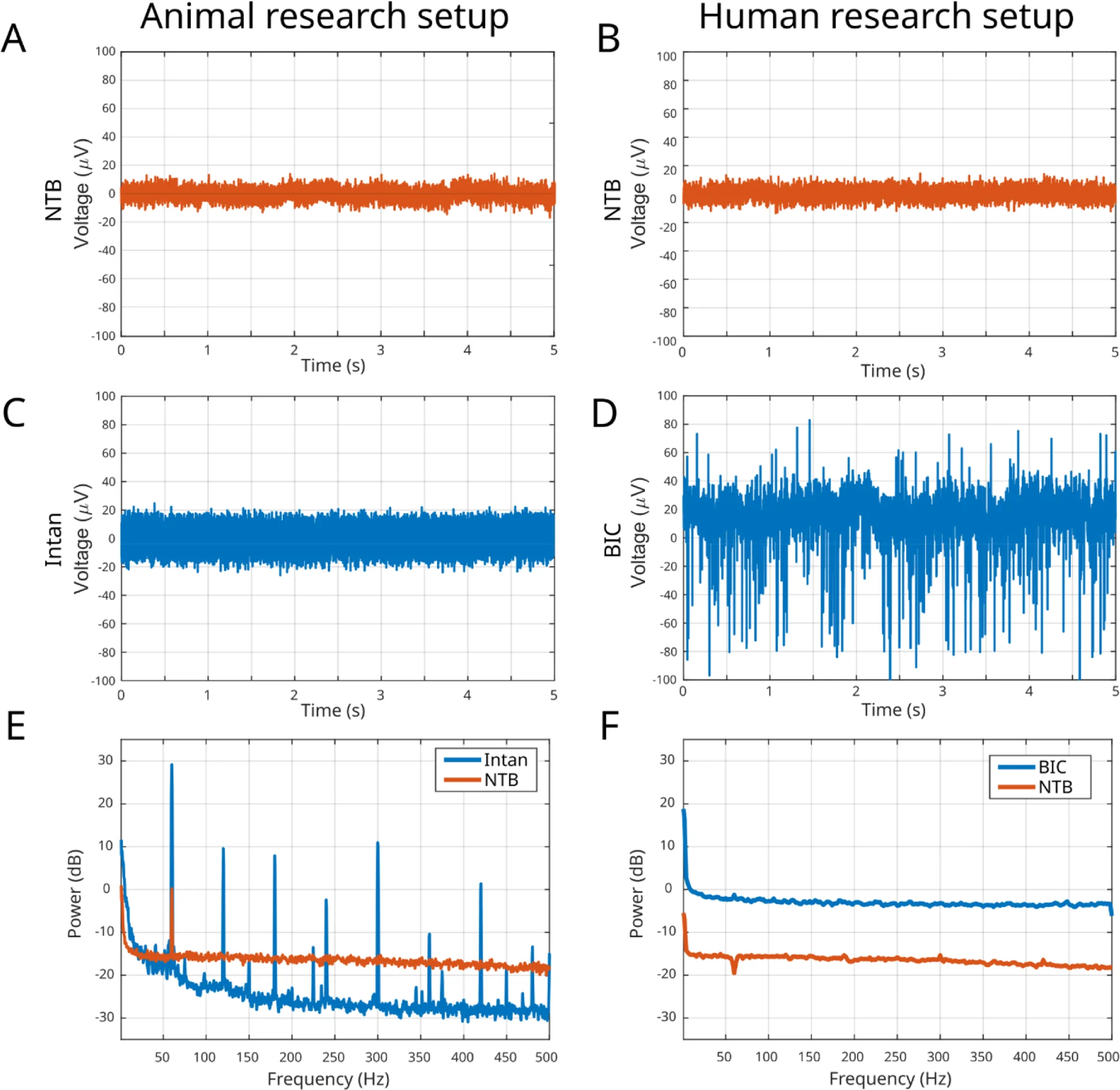
Noise floor characteristics of various data acquisition systems. Five seconds of data acquired from NTBs in saline using (A) the Animal-use research (AUR) environment and (B) the Human-use research (HUR) environment. (C) and (D) Recordings collected with an (C) Intan RHD2132 headstage via an Open Ephys DAQ and (D) CorTec BIC capturing the same activity depicted in (A) and (B), respectively. (E) Power spectral density plot comparing the spectral characteristics of the NTB and Open Ephys (Intan) data. While the Intan/Open Ephys data has less power at most frequencies, note the amplification of 60 Hz harmonics relative to the NTB. (F) Same as in (E) but comparing the NTB and the CorTec BIC. The BIC was found to have increased broad-spectrum noise relative to the NTB. With the exception of 60 Hz noise, due to differences in filter characteristics between the sites, the two NTB systems have nearly identical spectral characteristics (i.e. rapid exponential drop in power at low frequencies less than −15 dB of power at most frequencies).

### 3.3. Evaluating Neural playback

A key functionality of the NTB is its ability to play signals of interest (e.g. previously recorded neural activity) into saline. This neural playback functionality enables a benchtop alternative to *in vivo* experimentation and, for the case of the NTB, is augmented by the system’s recording functionality. Concurrent recording of benchtop activity enables the collection of ground truth data, providing a robust means to evaluate the recording capability of a system under test.

To assess the NTB’s biopotential generation and recording capabilities, electrophysiology data collected in both AUR and HUR contexts served as inputs to be played back by the system. Note, the data used in this manuscript was previously collected as part of other experiments that received approval from the IACUC or IRB of the relevant institution. Data for playback was re-sampled such that the sampling rate was 2 kHz before being written to a CSV file. We note that this output rate is excessive given the 1 kHz sampling rate used for the NTB in these experiments. Other users may choose any output rate from 500–2000 Hz (i.e. the programmatic default range of the NTB), though we recommend matching or exceeding the sampling rate to avoid staircasing in the acquired signal. The CSV file was then loaded into a microSD card to be read by the NTB. Calibration was also performed prior to initiating biosignal playback. After both the NTB and systems under test captured 1 min of playback (figure 4), cross-correlation was performed to compare the similarities between the original input and captured activity by the two systems.

**Figure 4.**
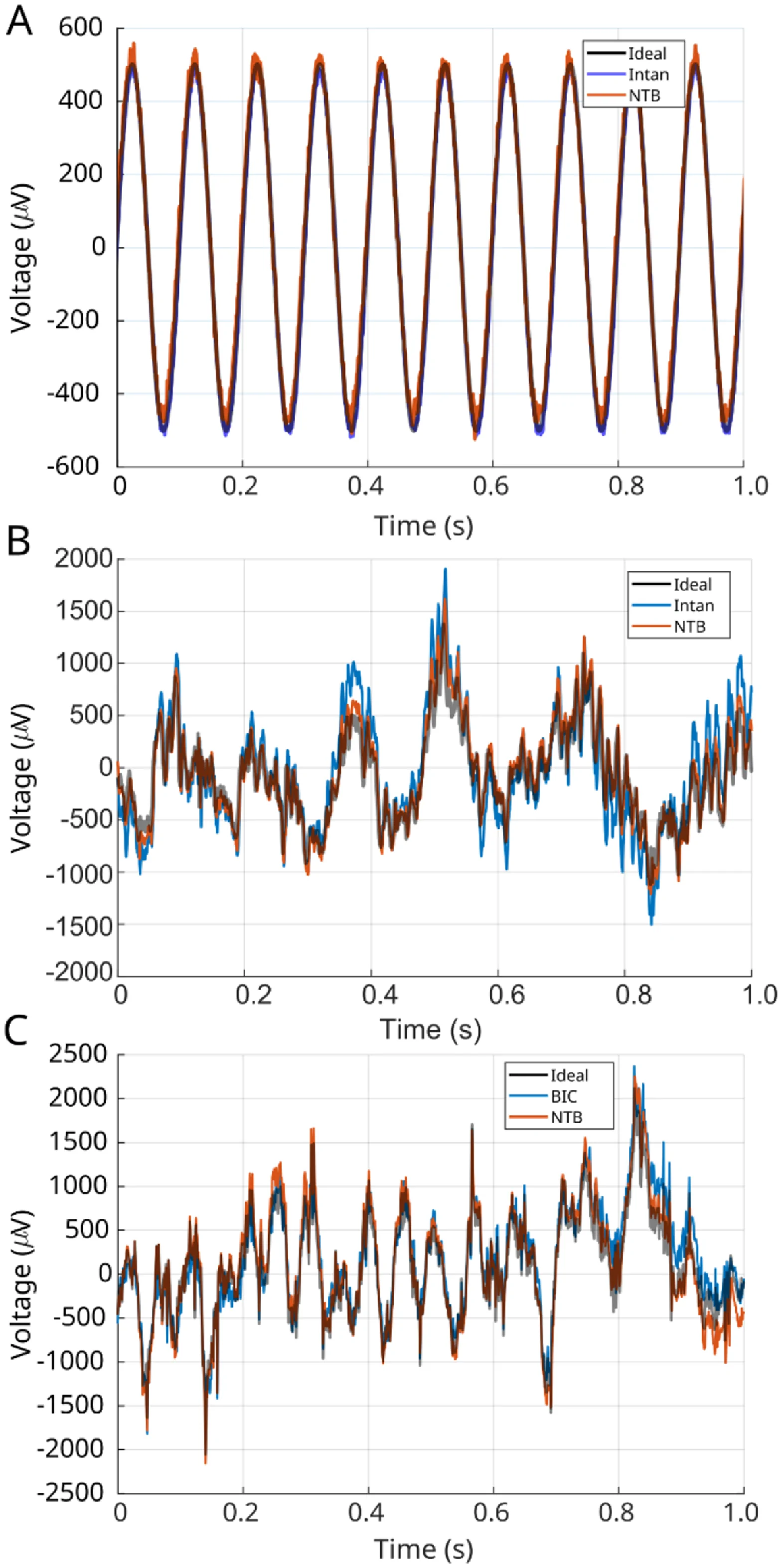
Demonstration of the current source waveform generator for playback functionality. (A) A 10 Hz, 1 mV peak-to-peak sine wave was generated by the NTB and injected into the saline tank for 1 s using the neural playback function. The input data (Ideal) is shown in black. The data recorded via the Intan RHD2132 (blue) and via the NTB (orange) is overlaid. (B) Neural activity collected from the rodent infralimbic cortex was also played back with the NTB and injected into the saline tank of the AUR testbench. One second of the recorded data is shown here comparing the ideal, Intan, and NTB data streams. (C) Neural sEEG data collected from a patient with epilepsy was similarly generated and injected into the saline tank of the HUR testbench via the NTB. One second of data comparing the Ideal, BIC (blue), and NTB (orange) is shown.

First, both sites generated CSV files containing a 10 Hz, 1 mVpp (i.e. one millivolt peak-to-peak) sine wave and injected this signal via the neural playback function as an initial assessment of how well the ACSWG of the NTB reproduces the input signal. Figure 4(A) shows data collected with the AUR environment. At both sites, the correlation between the input signal and the signal collected via the NTB and devices under test was very high (AUR: NTB *r* = > 0.99, Intan *r* = > 0.99; HUR: NTB *r* = > 0.99, BIC *r* = 0.96).

Next, we sought to test the ACSWG with more complex signals. For the AUR testbed, LFP data was collected from depth electrodes (Pt–Ir, 125 *µ*m diameter, <20 kΩ) in the infralimbic cortex of a rat during a free-behaving recording. Animals were placed inside a non-conductive container located inside a Faraday cage and allowed to freely explore the open space for the entirety of a five-minute recording. Electrophysiology data were routed through a Brownlee pre-amplifier (Model 440) to an OpenEphys DAQ and sampled at 30 kHz. These data were downsampled to 2 kHz and injected into the electrophoresis tank using the neural playback function (figure 4(B)). Data were routed to both the sensing portion of the NTB and to an IntanRHD2132 headstage using a splitter (described in the *AUR Benchtop Environment section* above). The correlation between the input signal and the NTB recording was *r* = 0.96, while the correlation coefficient for the input signal and Intan data was *r* = 0.87.

With the HUR testing environment, stereo-electroencephalography (sEEG) recordings collected during intracranial monitoring of a human participant with epilepsy were used for playback. These resting state recordings were originally collected with the CorTec BIC system connected to a subset of the implanted sEEG electrodes through externalized extensions ending in clinical touch-proof connections. From the original recordings, a select channel located in the motor cortex served as the input signal to be played back by the NTB. Due to packet loss from wireless communication, data was linearly interpolated to match the expected 1 kHz sampling frequency of the device prior to being injected into the saline tank for playback. Signal fidelity between the input and recorded signals was again high with the correlation coefficient between the original input and the sensed activity for the NTB being *r* = 0.95 while the BIC registered at *r* = 0.86 (figure 4(C)).

These data demonstrate the ability of the NTB to both generate and record complex signals while maintaining the integrity of the original dynamics of the input signal even when it is set up in non-idealized environments (e.g. mimicking a clinical setting where tight experimental control of the surroundings is not always possible).

### 3.4. EP simulation

The sensing circuit of the NTB is capable of communicating with the ACSWGs via the processing unit, which enables experimenters to generate reactions to specific stimuli. When the sensing circuit detects an event of interest, the processing unit can trigger the ACSWG to produce a pre-programmed response. While the user can set the specific parameters that define a triggering event, we initially programmed the NTB to detect stimulation pulses (i.e. large changes in energy at high frequencies) and react with a simulated EP that is defined by four customizable parameters to emulate an evoked response expected *in vivo*.

(1) The default amplitude range of the output is 1 *µ*V to 2000 *µ*V (figure 5(A)). The maximum of 2000 *µ*V can be overridden by the end user, however, the maximum voltage range will be limited by the test setup maximum impedance as the ACSWG can generate currents up to 10 mA with a maximum voltage range of ±14 V. Note, achieving an accurate voltage range requires use of the calibration function prior to EP testing.
(2) The latency of the EP following a detected event of interest (e.g. the stimulation artifact from an electrical pulse) can range from 0 to 1 s (figure 5(B)). The upper limit of this delay parameter can be programmatically adjusted.
(3) The frequency of the EP can be set between 1 Hz and 250 Hz (figure 5(C)). The cap of the frequency component can be increased programmatically by increasing the write speed of the DAC and changing the hardcoded frequency maximum.
(4) The final adjustable feature of the EP is the ‘template’ of the response. The NTB software currently supports three different EP templates to account for various EP morphologies (figure 5(D)): **Type I** features one full cycle of a sine wave at a single frequency in which the first half of the cycle is delivered at 100% amplitude and the second half of the cycle is delivered at 25% amplitude. The frequency and amplitude of the EP are set by the user. The sine wave has a negative leading phase, resulting in a starting voltage near 0 followed by a negative trough (figure 5(D), top). **Type II** consists of a cosine wave with a negative leading phase and the same amplitude decay as Type I (i.e. 100% for the first half cycle and 25% for the second half). This results in a positive voltage at EP onset that falls to a trough before moving to a lower amplitude positive peak (figure 5(D), middle). Finally, **Type III** features the combination of two sine waves at different frequencies set by the user. The first sine wave is delivered for one half cycle at 100% amplitude, while the second is delivered for a full cycle, with the first half at 100% amplitude the second half at 25% amplitude (figure 5(D), bottom). These template morphologies were modeled off of previous reports of cortical EPs collected from clinical populations [65, 88] and are intended to be a starting point rather than provide comprehensive coverage of EP characteristics.

**Figure 5.**
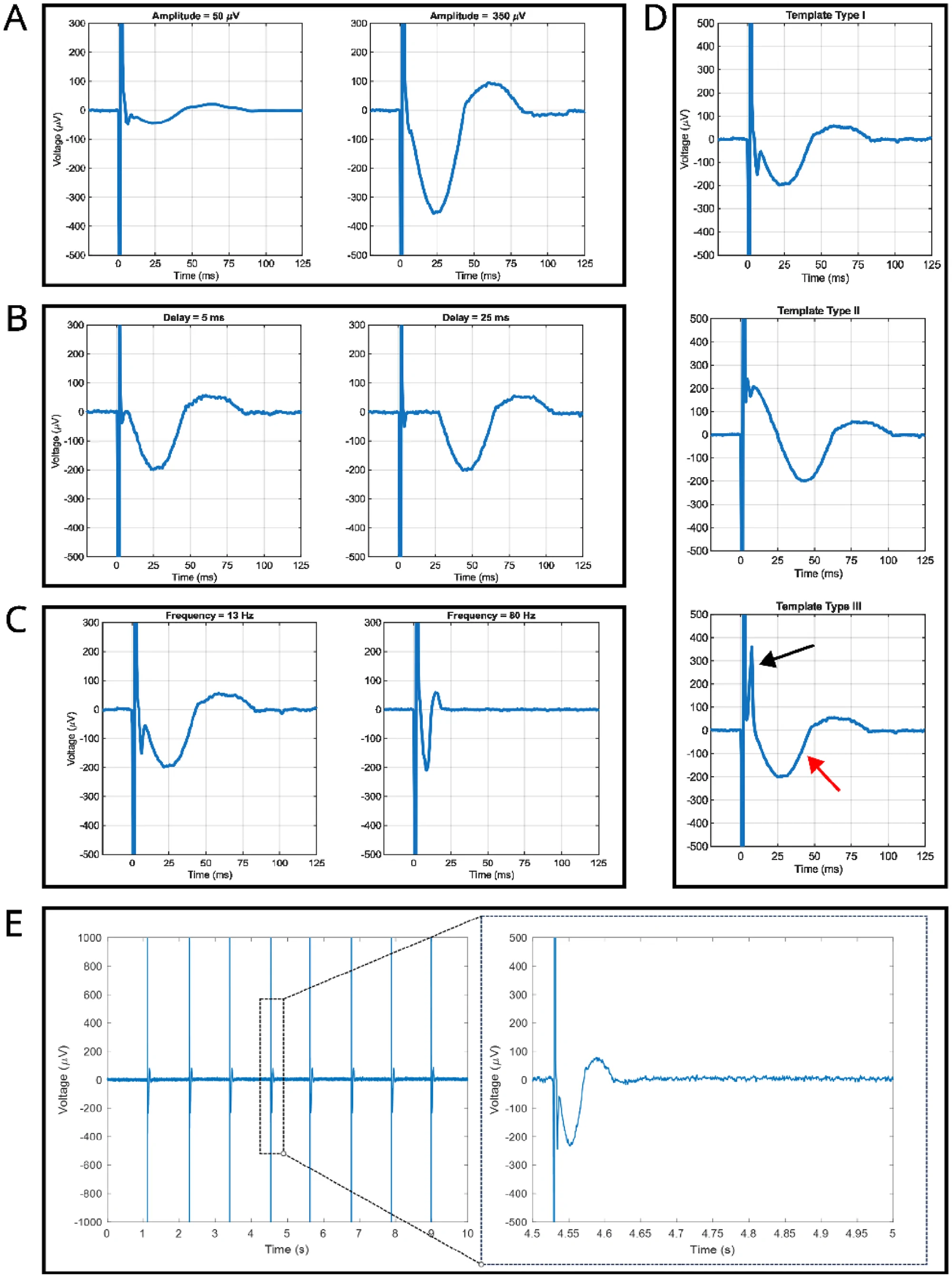
Configurable parameters for evoked potential simulation with the NTB. (A) Example of changing the amplitude component of the stimulated evoked potential (EP). Both EPs used a 13 Hz sine wave as a base. The left panel shows a 50 *µ*V EP, while the right shows a 350 *µ*V EP. (B) Example of changing the delay from stimulation detection to EP generation. Both EPs used a 13 Hz, 200 *µ*V amplitude sine wave as a base. The left side shows an EP delayed by 5 ms while the right shows a 25 ms delay. (C) Example of changing the frequency component of the EP. Both used a 200 *µ*V sine wave as a base. The left side shows an EP generated from a 13 Hz sine wave while the right shows an EP generated from an 80 Hz sine wave. (D) Example of changing the morphology of the evoked potential between three configurations. Type I—a negative leading sine wave in which the first half of the cycle is delivered at 100% amplitude, and the next half cycle is delivered at 25% amplitude. Type II—a single frequency, negative leading cosine wave where the first oscillation is delivered at 100% amplitude and the second half cycle is delivered at 25% amplitude. Type III—a leading high frequency, negative half sine wave (black arrow) followed by a low frequency full sine wave (red arrow) where the ratio between the two frequencies is 20 and the ratio between the two amplitudes is 2. The high frequency component is 100 Hz in this example. (E) Example trace depicting 1 Hz stimulation and the triggered simulated EP response over a 10 s time window (left) and a zoom-in of a single instance of stimulation artifact followed by a simulated EP signal (right). The longer time scale depicts no additional activity is generated with the EP simulation feature until another stimulation pulse is detected by the NTB.

In this stimulation context, the NTB’s data acquisition capability is particularly relevant, as stimulation can overwhelm a system’s amplifier, resulting in gaps of information with respect to the impact of stimulation on capturing evoked neural activity. By having ground-truth knowledge of the actual ‘biosignal’ in saline, the effects of stimulation and its artifacts can be characterized and not only inform the design of stimulation experiments but also guide signal processing methods for post-hoc analysis. Considering the variety of stimulation evoked activity, the NTB’s EP simulation functionality allows customization of the evoked response, providing an initial starting point for modeling biologically plausible EPs. We describe tests using this functionality below.

## 4. Applications of the neurotest platform

### 4.1. Evaluating recording configurations for EP experiments

We used the NTB to evaluate whether a simulated EP could be recovered from data collected via an Intan RHD2132 headstage during electrical stimulation artifacts. These headstages are common in preclinical and animal model-based electrophysiology applications but were not designed with electrical stimulation in mind (see the Intan RHS series for devices designed around stimulation experimentation). Despite this, several groups have reported electrical stimulation experiments using these chips with varying degrees of success at artifact mitigation (see [89–91] for a subset). This makes the RHD2132 chip an interesting edge-case: a device that appears able to tolerate stimulation experiments under certain conditions but one that lacks standardized or publicly available manufacturer testing data within this specific context.

To assess how quickly a known signal could be recovered following electrical stimulation when using an IntanRHD2132, we used an external stimulator (StimJim) to deliver biphasic stimulation pulses (±100 *µ*A, 100 *µ*s per phase) into the electrophoresis tank via a pair of platinum-iridium electrodes (∼4 kΩ impedances). This pulse was detected by the sensing portion of the NTB and a parallel data stream routed through an Intan RHD2132 to an Open Ephys data acquisition system. The simulated EP used the Template Type I format (i.e. produces one cycle of a 25 Hz sinewave where the first half of the wave is delivered at 100% amplitude and the next half at 25% amplitude). The sine wave was set to have a target realized amplitude of 150 *µ*V. In addition to being within a reasonable amplitude range of reported CCEP components [92, 93], this value was selected in order to ensure a detectable response that could also allow thorough assessment of the efficacy of post-hoc processing techniques to extract the underlying EP activity.

Delivery of electrical stimulation resulted in a large post-stimulation artifact in the Intan data stream that required ∼600 ms to settle back to baseline (figures 6(A) and (B)). To isolate the simulated EP, we employed a template subtraction technique. We found that the stimulation artifact present in the Intan data stream was not consistent, an issue present in some of our *in vivo* data (not shown) and discussed elsewhere (e.g. [33, 94]). This makes subtraction of a single template challenging, as no single template will match the shifting artifact. Instead, we created a series of artifact templates by delivering 20 stimulation pulses via the StimJim while the EP function of the NTB was disabled. By disabling the EP simulation function, we isolate a waveform that is solely driven by electrical stimulation artifact (i.e. artifact template). We then delivered sets of 5 stimulation pulses with the EP function active using either a 1 ms delay (figures 6(A),(C) and (E)) or 25 ms delay (figures 6(B), (D) and (F)) and saved the resulting data.

**Figure 6.**
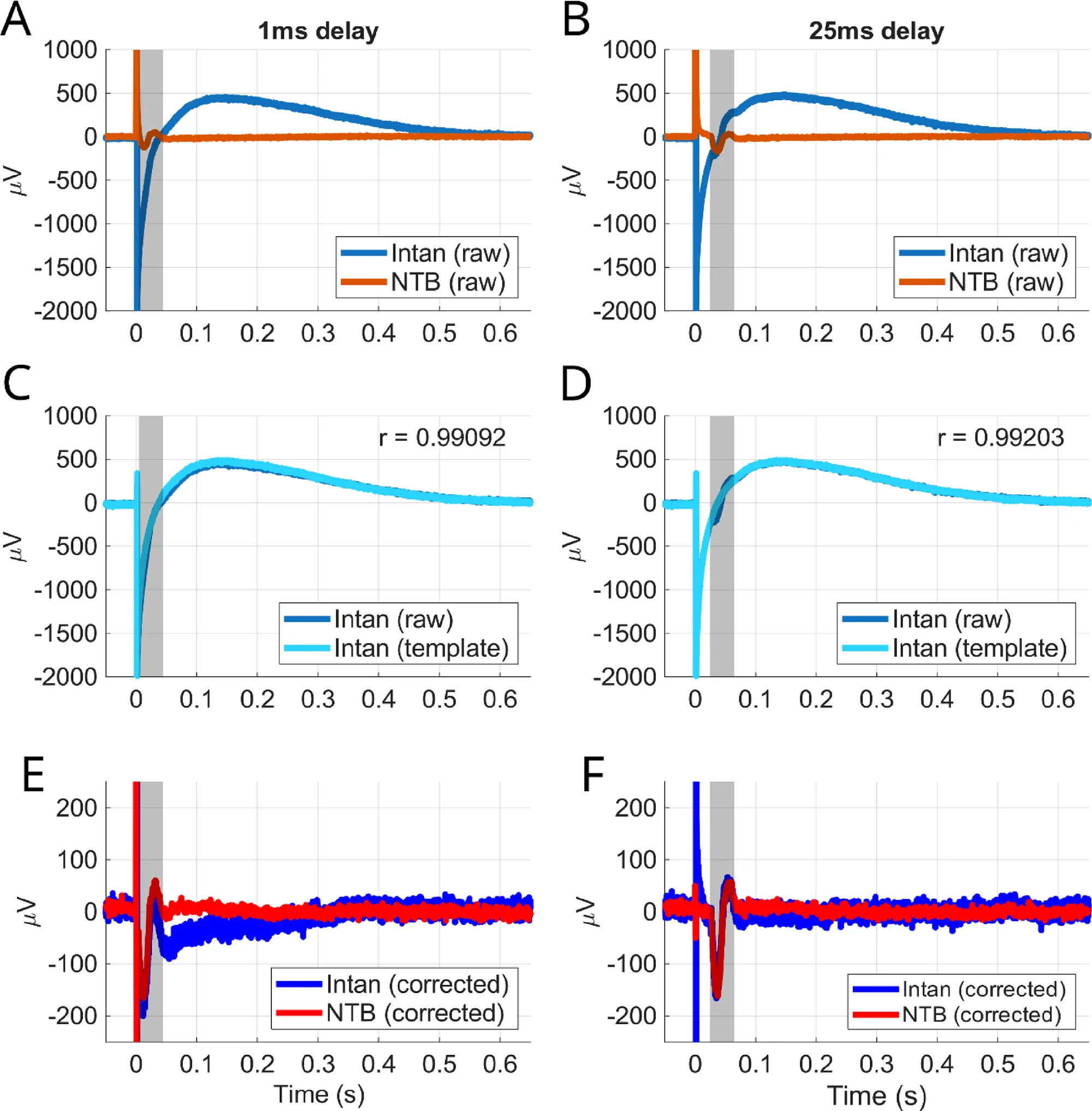
Using the NTB to develop an artifact mitigation approach based on simple template matching. (A) Raw time series data from the NeuroTest Board (NTB, orange) and Intan RHD2132 via Open Ephys (dark blue) setups depicting an example stimulation pulse and simulated evoked potential (EP). Gray shaded area shows the expected start and end times of the simulated EP (parameters: 25 Hz sine wave, 150 *µ*V target amplitude, Template Type 1, 1 ms delayed response relative to detection of stimulation pulse). Note the large post-stimulation artifact in the Intan data stream and the lack of a visually detectable EP. (B) Same as in A but using a 25 ms delay for the EP generation. (C) Same data from the Intan stream as shown in A but with a template matched artifact overlaid (light blue). The correlation coefficient between the artifact template and the raw artifact is displayed. (D) Same as in C but using a 25 ms delay. (E) Simulated EPs extracted from the NTB (red) and the Intan (blue) data streams using a template subtraction method (i.e. by doing a point-by-point subtraction of dark blue and light blue traces). (F) Same as in E but using a 25 ms delayed EP. Note that the voltage scale has been changed from A–D to visualize the precision of the amplitude of the EP following artifact rejection.

This data was then loaded into MATLAB and each of the five EP pulses was time-aligned with each of the 20 template artifacts. A timepoint-by-timepoint subtraction of the templates from the EP pulses was performed and the resulting difference was squared and summed. The result with the lowest value of this sum of squares was selected as the best fit template (example shown in figures 6(C) and (D)). We further verified accuracy of the template by calculating the correlation coefficient between the artifact template and EP pulse (as shown in figures 6(C) and (D)). In all cases, the correlation coefficients were *r* = > 0.99. Finally, we take the result of subtracting the best fit template from the raw data to show that the EP was recoverable (figures 6(E) and (F)) despite the large, inconsistent post-stimulation artifact present in the Intan data stream. Development of a similar template matching strategy purely using *in vivo* data containing stimulation artifacts of similar size and duration would be challenging as the known ground truth for the EP would not be available. However, this test verifies that the RHD2132 data stream does contain the EP embedded within the post-stimulation artifact, enabling future attempts at *in vivo* EP recovery.

### 4.2. Testbed for EP processing

We utilized the NTB’s EP simulation feature to assess the ability of the BIC to reliably capture stimulation evoked activity and evaluate the efficacy of post-hoc cleaning techniques for isolating the EP of interest collected via the BIC system. Using the recording configuration described previously, stimulation pulses were delivered by the BIC and triggered a consistent evoked response from the NTB. Stimulation was delivered once per second (1 Hz) to ensure that any post-stimulation artifact had ample time to settle before the next pulse was delivered. With the command interface, a 150 *µ*Vpp signal with 12 Hz frequency components (i.e. a pure, 12 Hz sine wave that followed the formula described in figure 5(D), Type I) was injected immediately upon detecting stimulation. This evoked response contained an initial negative peak around 25 ms post-stimulation, and a smaller positive peak about 60 ms post-stimulation. While these settings represent values that fall within normal ranges reported in EP literature [88, 95–97], we want to emphasize that these settings are programmable and easily changed to fit an end user’s needs. Throughout these stimulation experiments, both the BIC and NTB systems were set to record. For this characterization, 20 trials of stimulation were delivered with the BIC. During post-hoc analysis, the trials were epoched and then averaged to generate an average of the raw evoked response waveform.

Trial data and their raw averages from this stimulation experiment can be seen for the BIC and NTB systems in figures 7(A) and (B), respectively. The raw average computed from the BIC recordings shows the evoked response signal superimposed with an exponential decay as the amplifier recovered from stimulation. The much larger magnitude of the stimulation artifact and low-frequency nature of the return to baseline behavior made it particularly challenging to fully appreciate the underlying evoked response. With further inspection of individual trial outcomes, return to baseline behavior, particularly activity within 150 ms post stimulation, was highly variable, distorting the underlying EP response. Activity captured by the NTB shows the stimulation artifact generated by the BIC and the preserved components of the generated evoked response. Upon comparison between the NTB and BIC trials and averages, the variable exponential decay from stimulation was unique to the BIC dataset only, indicating that this phenomenon is specific to the BIC device. Previous work utilizing stimulation with the BIC device [84] has shown similar exponential decay trends and presents an ongoing signal processing challenge for acquiring underlying signals of interest.

**Figure 7.**
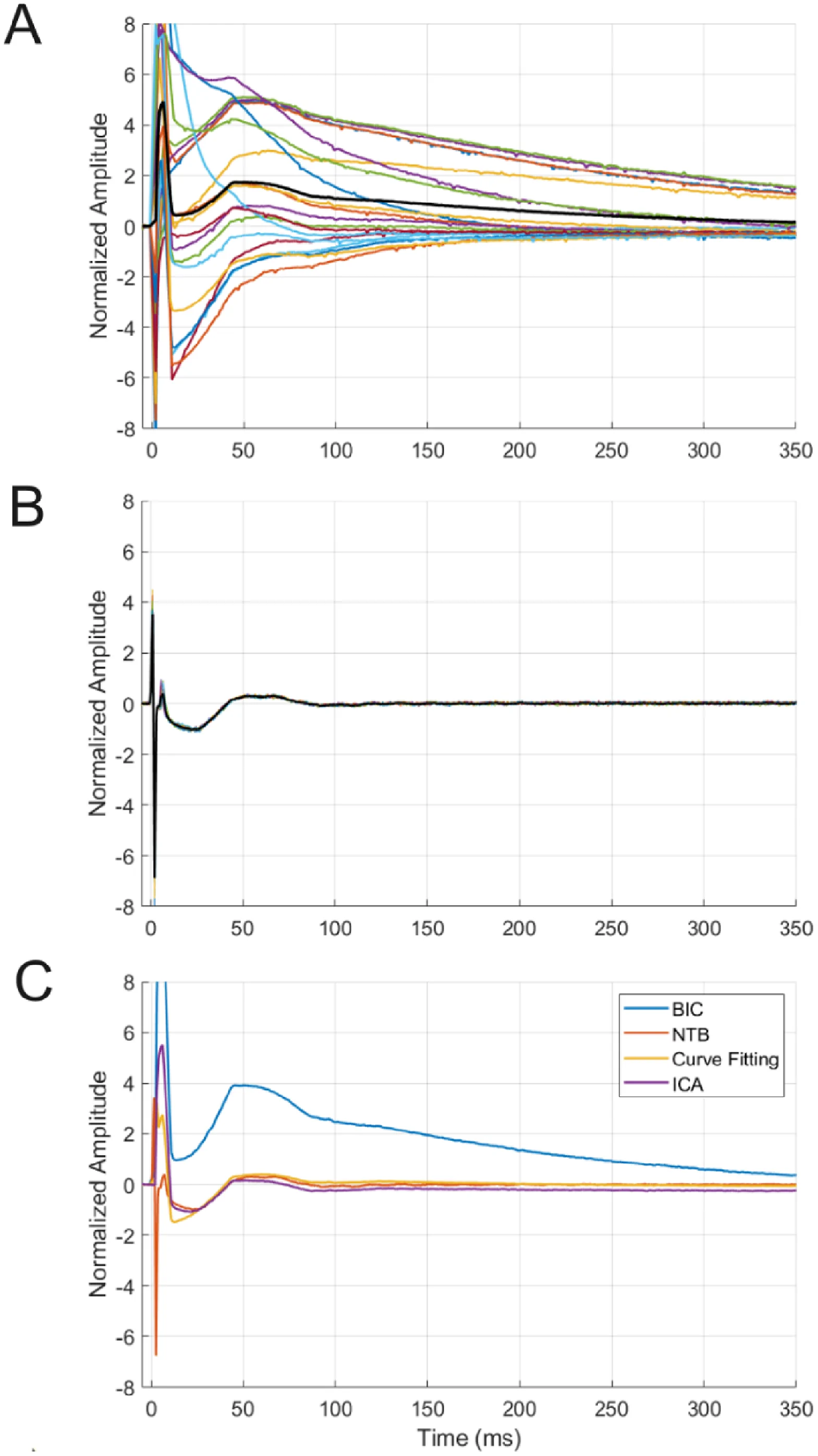
Example case of using the NTB and its evoked potential functionality to assess signal processing methods. Time synchronized plots of normalized, simulated evoked potentials (parameters: 12 Hz, 150 *µ*V, Template Type 1, no delay) as captured by (A) the CorTec Brain InterChange (BIC) and (B) the NeuroTest Board (NTB). Colored traces show individual trial data while the raw average of the trials is plotted in black. These colored traces are obscured in panel B due to complete overlap. (C) Outcomes from applying independent components analysis (ICA) and curve fitting approaches to target the return to baseline behavior are illustrated and compared to the ground truth recorded by the NTB and the original average response from the BIC. Both signal processing approaches targeted the low-frequency return to baseline observed in the original BIC trace. Normalized amplitudes facilitated comparison of cleaned responses between signal processing techniques.

Because of interest in the temporal characteristics of evoked activity, we implemented different strategies to minimize the impact of the exponential trend on the evoked response. In this application, we applied a curve-fitting approach and independent component analysis (ICA) to clean the BIC dataset. To facilitate comparisons between these methods, signal outcomes from these cleaning methods, Y_C_, were normalized. For assessing the efficacy of these methods to extract the underlying evoked responses, the NTB outcomes, Y_O_, were used as an effective ground truth dataset to compute the relative error of the cleaned signals. Relative error was calculated with the following equation where || *x* ||_2_ denotes the Euclidean norm of the signal, *x*:

\begin{equation*}Relative\;Error = {\left\| x \right\|_2} = \frac{{{{\left\| {{y_c} - {y_o}} \right\|}_2}}}{{{{\left\| {{y_o}} \right\|}_2}}}.\end{equation*}

We first utilized the curve-fitting approach. The average response from utilizing this method can be seen in figure 7(C). To target the exponential trends captured during these stimulation experiments, an exponential curve was modeled to the overall trend of the trial before being subtracted from the trial data. This method towards detrending was somewhat effective towards better realizing the evoked response. While this approach helped offset the exponential trend, it did not fully recover all components of the simulated response, in particular, activity that occurred during or shortly after stimulation. Additionally, it was noted that poor fits can result in the creation of pseudo-oscillations that could be mistakenly interpreted as electrophysiological activity or distort the underlying oscillations of interest. Considering that the underlying EP is an unknown signal, an additional vetting process should be incorporated to ensure the quality of a fit prior to detrending. However, it should be noted that while metrics such as root mean square error could potentially mitigate the application of poor fits to detrend data, it may be difficult to determine the threshold to discern a poor fit due to the presence of underlying activity. In using this curve-fitting approach, the relative error from utilizing this method was computed to be 50%.

Another method applied was ICA. Prior efforts have utilized ICA for targeting artifacts and their distortions in various stimulation evoked response experiments [98, 99]. Using the FastICA method [100], ICA components were selectively removed to eliminate the stimulation artifact recovery period overwhelming the underlying EP response. Because of the low-frequency nature of the return to baseline behavior and possible overlap with the lower frequency nature of the simulated signal, components were manually identified prior to removal. This approach resulted in reasonable recovery of the components of the simulated response, as depicted in figure 7(C). Compared to the curve-fitting outcomes, application of ICA allowed the recovery of the negative peak 25 ms post-stimulation. However, it should be noted that this approach must be customized to each dataset, as evoked activity can be composed of various neural sources. With ICA, the relative error was determined to be 36%. Outcomes with the ICA approach resulted in slightly improved recovery compared to the curve-fitting approach; however, evaluation of additional processing methods is of interest in order to yield further improvements in signal recovery. While a thorough investigation of artifact mitigation techniques is beyond the scope of this work, these results demonstrate that the NTB can be used to facilitate these tests.

## 5. Discussion

In this work, we present the NTB, a controlled, benchtop evaluation system that enables extensive device characterization and validation to guide neuromodulation research efforts. The NTB and electrophoresis tank provide a platform that is designed to mimic bio-electric signals as well as the tissue-electrode interface, allowing for *in vitro* alternatives to some aspects of *in vivo* testing. Assessments across labs demonstrated highly consistent performance of the NTB despite distinct environments, including differences in the devices tested for data acquisition (i.e. Intan RHD2132 and CorTec BIC). Noise floor characteristics and signal generation fidelity of the NTBs were nearly identical, establishing a reliable benchmark for assessing functionality of other devices. We further provide two separate use-cases of the neural playback functionality and assess stimulation artifacts in simulated EP experiments. Taken together, these data demonstrate that the NTB can be used for critical testing and validation of brain stimulation and sensing technologies across sites, devices, and applications.

Reproducibility is critical for scientific research [101–104] and the need for reliable benchmarks will only increase as electrophysiology experiments and related technologies become more complex. This reality is the core motivation for the creation of open-source devices like the NeuroDAC [35] and our NTB. Having systems that can be used flexibly across many different applications and experimental setups allows for comparison across projects with disparate methodologies but overlapping scientific purposes. In our case, the teams at each site have similar end goals (i.e. developing new neuromodulation therapies for neurological and neuropsychiatric disorders); however, the setup at the first site was intended to model preclinical research that uses animal models while testing in the second site modeled a use case in clinical research. The first team used electrodes, stimulators, and data acquisition systems common in animal model-based research while the second team worked with a neuromodulation device intended for HUR. Despite these differences in equipment and target applications, we demonstrate that the NTB behaves predictably and similarly across these two laboratory setups despite their different methodological approaches.

The noise floors on our NTB devices qualitatively follow expected trends, such as that of power-law scaling, starting around 0 dB and bottoming out around −25 dB. General background noise in both systems was ∼4 *µ*V RMS despite the lack of faraday cages or other electrical isolation and environmental noise mitigation techniques often present in electrophysiology rigs. This noise floor is similar to that reported with other comparable non-commercial devices [35]. Given that neural signals recorded via intracranial electrodes generally have amplitudes in the 1–500 *µ*V range, these data show that the NTB can reliably detect even small simulated biopotentials. We further demonstrate that the signal generator of the NTB exhibits extremely high-fidelity replay capabilities both with simple signals (i.e. sine waves) and complex neural data from both rodents and humans. While we do not evaluate with recordings from an expansive set of electrodes, we believe that our initial evaluation, combined with additional testing, will demonstrate that the NTB is capable of faithfully generating activity regardless of electrode modality. These data highlight the potential for this system to be used across the translational pipeline, from preclinical animal modeling to evaluation of investigational devices.

We opted to use a current-source over a voltage-source for the arbitrary waveform generator portion of the NTB due to the relative increase in stability and predictability of signal output from these systems in environments with unknown or varying impedances. Voltage-source systems must adjust output current as the resistive load changes in order to maintain a constant voltage level; however, a current-source system drives a constant current output regardless of changes to load impedance (provided the output impedance of the system is much higher than the load impedance). The impedance of biological systems and implanted electrodes can change on various time scales as processes such as tissue reaction to electrode implantation [105], capacitive changes at the electrode-tissue interface during stimulation [106], or as natural intrinsic fluctuations [107] occur. Although there are circuit architectures that can mitigate this issue in voltage-source systems (as noted in [35]), a current-source system with high output impedance is generally more appropriate as a base architecture when the device under test is intended for use in an electrolyte medium [108, 109]. These issues are part of why modern DBS systems and other IPGs for neural stimulation have largely been encouraged to move away from voltage-controlled generators [110].

A core use case of the NTB is emulating EPs. This functionality allows us to conduct experiments assessing the impact of stimulation artifacts and device amplifier limitations on known input signals (i.e. the simulated EPs). We used this functionality to show that the commonly used Intan RHD2132 digital headstage could reliably be used to record simulated EPs within 1 ms of a large stimulation artifact under our specific testing conditions. The large amplitude and rapid exponential/non-linear decay of the post-stimulation artifact makes assessing any underlying physiology difficult, particularly in an *in vivo* context where a neural evoked response may or may not be present. The inconsistency of the post-stimulation artifacts further complicates any *in vivo* assessment. Here, we leveraged two main components to assess signal fidelity: (1) the parallel data stream routed to the NTB, which remains largely free from post-stimulation artifacts and (2) the known ground truth of the simulated EP. We provide a simple methodology for artifact removal in benchtop testing that can be validated against these two components. This approach allows users to find the necessary conditions to recover neural data post-stimulation as rapidly as possible without the need for complicated and uncertain *in vivo* testing. We note that mitigation of stimulation artifacts via algorithmic template matching approaches is a common way to clean data (e.g. [31, 111–113]) and that the NTB or other benchtop tools are not required for developing this methodology. Rather, the NTB expedites and simplifies development of these strategies by providing a well-controlled, low-cost testbench for assessing algorithm performance. Lastly, we want to again note that we used the RHD2132 due to its ubiquity and because our NTB testing application represented an edge case for use of this equipment. The Intan RHS series is an alternative system purpose-built for electrical stimulation experiments.

In our HUR testbed, we were able to assess the post-stimulation artifacts observed with the CorTec BIC. With a lower sampling frequency and more convoluted return to baseline behavior, the BIC system requires more advanced post-hoc techniques to extract the underlying response of interest. Simulations of CCEP experiments with the NTB allowed us to generate a data set to evaluate analytical techniques for artifact removal. In both sets of tests, outcomes from the cleaning methods were compared to the known generated outputs captured by the NTB. Assessments with this ground truth dataset identified nuances in these processing methods and set up an initial pipeline that can be applied when approaching *in vivo* CCEP collection with the BIC device.

The tests described in this manuscript are not intended to be a comprehensive list of possible scenarios that can be addressed with the NTB. Other factors that may impact charge delivery, signal acquisition, or the profile of stimulation artifacts include the size, materials, and relative positions of stimulating or recording electrodes. Electrode array form factors, the stimulation waveform or pulse generator used to deliver charge, and other characteristics of implantable devices will also influence these variables. Interactions between third party equipment and the device under test can also introduce troublesome noise. We also acknowledge that we only present a limited number of EP waveforms here and that different groups may be interested in specific EP characteristics beyond our original scope. We hope that the open-source nature of the NTB will encourage researchers and clinicians to collect, test, and report data on a myriad of factors affecting the performance of implantable devices.

We want to note that many other *in vitro* test benches for assessing electrophysiological equipment exist already and benchtop testing is commonly used as part of regulatory approval processes (e.g. [114]). A particularly relevant system by Powell *et al* 2021 [35] is described in our work, which provides details on an open source biosignal waveform generator, called the NeuroDAC. This system was used to test performance of the Summit RC + S device from Medtronic. The NeuroDAC has many great design features but does not have the EP functionality presented here and was initially designed to play signal into resistive circuits (though it can function with devices being tested in saline). Prior work by Haci *et al* 2017 [41] detailed a benchtop biosignal generator, but the system also lacks an EP function, is not open-source, and was not built for tests in an electrolyte medium. We also note that proprietary systems are available as well. Companies such as Blackrock Neurotech, Plexon, and Tucker Davis Technologies make benchtop tools for testing recording systems without needing connection to a live subject [38–40]. While these tools offer great ease of use and come with costumer support, they are proprietary (i.e. not open source), can be limited in the devices they can interface with (e.g. requiring specific omnetics or samtech connectors), and are not capable of adaptive signal output.

One set of tools we have not yet discussed are *in vitro* systems that make use of cell culture assays to model biological systems, e.g. [95], including modeling of evoked activity in a much more controlled environment relative to *in vivo* testing. Although these systems provide high biological validity, the need for cell culture expertise, greater control of the testing environment (e.g. pH, CO^2^), auxiliary equipment (e.g. specific multi electrode arrays, MEAs), and relatively high price (>R50 000, e.g. [115, 116]) limits the broader accessibility of these tools. Some groups have created open-source alternatives to these commercial products [117] that help decrease cost and increase accessibility. However, these types of tools may still not be an option for smaller groups, particularly startups to mid-sized companies or academic labs without access to cleanroom fabrication facilities or without cell culture expertise. Another limitation is that these tools are traditionally 2-dimensional (i.e. cells grown on a single, planar surface). Interfacing implantable neurostimulators, such as DBS leads, with these 2D systems is a challenge, however, exciting advances in 3-dimensional cell culture technology [118–120] may mitigate this issue in the future.

Ultimately, end users should take care to select the testing tool that is optimally fit for purpose according to their specific needs. The NTB offers an accessible tool for sensing and emulating biopotentials. It enables testing of systems reliant on stimulation EPs, including artifact mitigation and EP recovery techniques. The simple design ensures that the NTB can interface with a wide array of neuromodulation devices, including implantable neurostimulators. It is built around testing in electrolyte mediums and allows for maximum flexibility in neural signal emulation while maintaining safe detection of large amplitude stimulation artifacts. The open source and low cost of the NTB enable broad accessibility and potential for future collaborative growth.

Although the presented NeuroTest platform expands on current biopotential emulation systems, there are notable limitations with the NTB system. Currently, the NTB only supports two input and output channels, placing a limit on the complexity that can be emulated in this testing environment. This configuration enables testing of many important issues such as a system’s susceptibility to noise (figure 3), ability to detect responses to stimulation, and single-channel artifact mitigation strategies (figures 6 and 7). It is intended that end users wire the NTB to individual pins on the device under test rather than test whole arrays simultaneously. This reduces the need to create a suite of device specific connectors. However, this setup may not be appropriate for emulating more complex environments [4, 121, 122], particularly within applications exploring multi-source brain activity (e.g. cross frequency coupling or coherence [123, 124]) or investigating spatio-temporal sensing configurations (e.g. travelling waves [125] or topographical functional mapping [126–128]). Expansion of the current circuitry is relatively straightforward, though, and would mainly require duplication of the existing architecture (i.e. expanding the printed circuit board to include more sensing ports). This expansion would enable testing simultaneous testing of all electrodes across larger recording arrays, however, simultaneous modeling of activity from distinct brain regions will require further development of the saline tank environment. Currently the tank is a single contiguous electrolyte medium, meaning that signals generated across cathode and anode of the tank are registered approximately the same on electrodes of similar impedances. Another shortcoming of the present NTB is the use of a microSD to load data available for playback. While advances in memory technology have enabled significant increases in storage capacity, the process of modifying data for playback, reuploading the data to the memory card, and then physically mounting the card to the NTB can be laborious and serve as an inconvenient barrier for rapid iteration through various signals for testing. One alternative is to allow direct signal loading via serial commands, improving flexibility with the device that can be enabled with firmware and software development.

Additionally, while the NTB can support a range of signals for playback, emulation of spiking activity is limited by the settling time of the DAC which is 1 *μ*s. Likewise, the ADC sampling rate is set to 1 kHz by default, meaning that this device is currently optimized for applications involving meso- to macro-scale neural activity (e.g. local field potentials). However, increasing the maximum speed of the signal generator is possible through removal of the feedback capacitor, while the ADC sampling rate can be programmatically set to higher frequencies (up to 32 kHz). These adjustments could be made to enable the high temporal precision needed for fine grained emulation of action potentials, though further testing would be warranted. We would also like to note that the NTB is not currently intended for use within slice electrophysiology experiments, which may require more sensitive electronics or lower noise floors on sensing components [129]. We are currently developing the NTB for use in behavioral electrophysiology rigs or clinical settings; however, because the system is open-source, end users are free to adapt the designs for use in more sensitive settings. Lastly, we would like to note that we intend for the NTB to supplement and improve *in vivo* testing, not replace it. We view this device as serving the Refine and Reduce goals of the 3Rs [130, 131], as modelling the full complexity of the *in vivo* systems we are attempting to emulate is beyond our capabilities.

In the future, the neural playback and EP functionalities of the NTB can be used for more sophisticated assessments of closed-loop algorithms and experimental designs. While we showed how the NTB reacts to detected electrical stimulation pulses in this current work, a future end user could update the NeuroTest code to react to a particular signal of interest (e.g. the increases in power in the beta frequency band often associated with bradykinesia in Parkinson’s Disease [132]). Likewise, the NTB could be used to generate more complicated responses than brief EPs. A user could program the system to respond to a detected signal of interest by injecting a persistent increase in band-specific power or input signal from a different neural state. Additionally, we plan to update the software of the NTB to allow concurrent use of the EP and neural playback functionality in order to enable better modeling of *in vivo* stimulation conditions (i.e. generate known mixtures of neural response and stimulation artifact). The open-source code could also be updated to allow for stimulation parameters that adapt over time, or the system could be programmed to cycle through evolving simulated neural states to mimic neuroplastic changes over time. Lastly, there are ample possibilities for improving the physical testing environment. Customization of the saline tank into discrete, partially connected chambers could enable emulation of multiple brain networks simultaneously. The electrolyte medium could be further upgraded into a full brain phantom (e.g. [133]) to provide even better emulation of the tissue-electrode interface and physical properties of the brain. These advances would enable testing of cutting edge adaptive neuromodulation technology (e.g. [12]). We intend to continue to improve the NTB by addressing the hardware and software limitations noted above in order to facilitate experiments that feature embedded neuronal models, multi-source/network level interactions, and optimization of stimulation parameters in implanted neuromodulatory systems [46, 94, 134, 135].

## 6. Conclusion

With advances in the neuromodulation space, the need for evaluation tools will play a significant role in ensuring adequate preparation before starting *in vivo* research. In building off of currently available evaluation systems, there will be more accessible means to assess electrophysiology equipment and experimental setups. Better informing users of the nuances of the systems they are utilizing in novel research efforts will guide upcoming experiment design and facilitate improved hardware design for future equipment. By expanding the resources available to perform system evaluation work, researchers can raise confidence in the novel systems they are utilizing and support more rapid and reliable prototyping efforts within the neural interface and neuromodulation space.

## Data Availability

The data that support the findings of this study are openly available at the following URL/DOI: https://github.com/uw-herron-lab/NeuroTest-Board [136].

## Supplemental material

Code and design files for this work will be available at the following repository following publication of this article: https://github.com/uw-herron-lab/NeuroTest-Board.
